# Simplified prognostic model for critically ill patients in resource limited settings in South Asia

**DOI:** 10.1186/s13054-017-1843-6

**Published:** 2017-10-17

**Authors:** Rashan Haniffa, Mavuto Mukaka, Sithum Bandara Munasinghe, Ambepitiyawaduge Pubudu De Silva, Kosala Saroj Amarasiri Jayasinghe, Abi Beane, Nicolette de Keizer, Arjen M. Dondorp

**Affiliations:** 1 National Intensive Care Surveillance, Quality Secretariat Building, Castle Street Hospital for Women, Colombo 08, Sri Lanka; 2 Mahidol Oxford Tropical Medicine Research Unit, Faculty of Tropical Medicine, Mahidol University, 3/F, 60th Anniversary Chalermprakiat Building, 420/6 Rajvithi Road, Bangkok, 10400 Thailand; 3 Network for Improving Critical Care Systems and Training, 2nd Floor, YMBA Building, Colombo 08, Sri Lanka; 4 Intensive Care National Audit & Research Centre, No. 24, High Holborn, London, WC1V 6AZ UK; 5 Department of Clinical Medicine, Faculty of Medicine, University of Colombo, No. 25, Kynsey Road, Colombo 08, Sri Lanka; 6 Academic Medical Center, University of Amsterdam, Meibergdreef 9, 1105 AZ Amsterdam-Zuidoost, Netherlands

**Keywords:** Critical care, Prognostic model, Model performance, APACHE II, Lower middle income country, Resource limited settings

## Abstract

**Background:**

Current critical care prognostic models are predominantly developed in high-income countries (HICs) and may not be feasible in intensive care units (ICUs) in lower- and middle-income countries (LMICs). Existing prognostic models cannot be applied without validation in LMICs as the different disease profiles, resource availability, and heterogeneity of the population may limit the transferability of such scores. A major shortcoming in using such models in LMICs is the unavailability of required measurements. This study proposes a simplified critical care prognostic model for use at the time of ICU admission.

**Methods:**

This was a prospective study of 3855 patients admitted to 21 ICUs from Bangladesh, India, Nepal, and Sri Lanka who were aged 16 years and over and followed to ICU discharge. Variables captured included patient age, admission characteristics, clinical assessments, laboratory investigations, and treatment measures.

Multivariate logistic regression was used to develop three models for ICU mortality prediction: model 1 with clinical, laboratory, and treatment variables; model 2 with clinical and laboratory variables; and model 3, a purely clinical model.

Internal validation based on bootstrapping (1000 samples) was used to calculate discrimination (area under the receiver operating characteristic curve (AUC)) and calibration (Hosmer-Lemeshow C-Statistic; higher values indicate poorer calibration). Comparison was made with the Acute Physiology and Chronic Health Evaluation (APACHE) II and Simplified Acute Physiology Score (SAPS) II models.

**Results:**

Model 1 recorded the respiratory rate, systolic blood pressure, Glasgow Coma Scale (GCS), blood urea, haemoglobin, mechanical ventilation, and vasopressor use on ICU admission. Model 2, named TropICS (Tropical Intensive Care Score), included emergency surgery, respiratory rate, systolic blood pressure, GCS, blood urea, and haemoglobin. Model 3 included respiratory rate, emergency surgery, and GCS. AUC was 0.818 (95% confidence interval (CI) 0.800–0.835) for model 1, 0.767 (0.741–0.792) for TropICS, and 0.725 (0.688–0.762) for model 3. The Hosmer-Lemeshow C-Statistic *p* values were less than 0.05 for models 1 and 3 and 0.18 for TropICS. In comparison, when APACHE II and SAPS II were applied to the same dataset, AUC was 0.707 (0.688–0.726) and 0.714 (0.695–0.732) and the C-Statistic was 124.84 (*p* < 0.001) and 1692.14 (*p* < 0.001), respectively.

**Conclusion:**

This paper proposes TropICS as the first multinational critical care prognostic model developed in a non-HIC setting.

**Electronic supplementary material:**

The online version of this article (doi:10.1186/s13054-017-1843-6) contains supplementary material, which is available to authorized users.

## Background

The burden of critical illness in lower- and middle-income countries (LMICs) and the growing urgency to improve outcomes are global priorities. If basic public health needs were better addressed, improvements in curative care, in particular for the critically ill, would be increasingly feasible [1]. The increase in availability of intensive care units (ICUs) in LMICs is a reflection of this phenomenon [2, 3]. Monitoring ICU performance by establishing medical registries and improving opportunities for healthcare training in LMICs are part of diverse attempts to improve the relatively poor outcomes for patients in such settings [4]. Critical care facilities in resource-limited settings, for example bed ratios, equipment, staffing, and skills, vary widely both within and between countries within South Asia. For example, in Sri Lanka ICU beds make up 1.3% of total beds, whilst in India, Bangladesh, and Nepal the proportion ranges from 5–8%, 4.8%, and 4.7%, respectively [2, 5–8] Critical care prognostic models such as the Acute Physiology and Chronic Health Evaluation (APACHE) and the Simplified Acute Physiology Score (SAPS) are in widespread use, especially in high-income countries (HICs). These models are used for risk stratification of critically ill patients, benchmarking of ICUs, recruitment of patients to clinical trials, and gauging the response to interventions. APACHE II and SAPS II are the most widely used, especially in LMICs (Haniffa et al., unpublished data), and are often used as a reference for the evaluation of other models.

Current critical care prognostic models are predominantly developed in HICs and require validation prior to implementation in LMICs. Studies validating and customizing existing models in LMICs are limited and come mostly from single centres using relatively small datasets [9–12]. Disease epidemiology, case-mix, and pathology, in addition to varying availability of resources necessary to diagnose, monitor, and treat critical illness, vary greatly both between resource-limited settings and when compared to HICs [2, 5–8]. Furthermore, the absence of accessible electronic patient information systems and devices may challenge the feasibility of performing probability calculations, for example when recruiting to research studies or for triage.

In an effort to overcome these barriers and the challenges of missing data, contemporaries developing models for LMIC settings are evaluating the performance of single-parameter scores, and those which prognosticate in the presence of infection (sepsis) [13, 14]. However, these too are based on single centre studies and their generalisability or adoption in clinical practice has yet to be evaluated.

We have previously reported on the limitations of the APACHE II model in ICUs in a LMIC, partly due to the incompleteness of data, which cannot be adequately overcome by the use of imputation techniques [15]. This difficulty and the need for further research and development of setting adapted models have been previously highlighted [16].

This study proposes a simplified critical care prognostic model for use in resource-limited settings and beyond, derived from a large South Asian ICU dataset. The proposed prognostic model aims to cover all diagnostic categories admitted to ICU, be applicable in resource-limited settings with a limited number of commonly available measurements, and be useable with the aid of a nomogram to calculate an approximate probability of survival without recourse to electronic equipment.

## Methods

This was a prospective cohort of patients admitted to the ICU (aged 16 years or older). Outcome was described as survival or nonsurvival at discharge from the ICU. The datasets leveraged in this study were derived from one ICU in India, one in Nepal, one in Bangladesh, and 18 ICUs in Sri Lanka. Part of the dataset from Sri Lanka was previously used to assess the applicability of the APACHE II model and is from the period prior to the establishment of a national critical care registry [15, 17]. Datasets from the other three countries were generated during the evaluation of the impact of a modular ICU training program in South Asia [18]. Data were collected prospectively at all sites and measures were taken to train the data collectors to ensure consistency, as previously detailed [15, 18]. A waiver for ethical approval including waiver of consent was obtained from the Oxford Tropical Medicine Research Ethics Committee (OXTREC) [18]. For the Sri Lankan dataset a waiver for ethical approval including waiver of consent was obtained from the Ethics Review Committee of the University of Colombo Faculty of Medicine.

The datasets from the four countries were combined to increase generalizability for the South Asian setting. Disparate paper systems along with limitations in study resources meant that following patients until hospital discharge in this setting was not feasible. For this reason, ICU discharge status was used.

All patients aged 16 years or over were included.

### Selection of variables

Selection of candidate covariates for model development was based on their use in existing case-mix prediction models, perceived clinical importance, and the feasibility of measurement in the settings of the participating ICUs. The variables included patient age, type of admission (elective surgery, emergency surgery, or medical) as per APACHE II coding [19], and admission clinical assessments of temperature, heart rate, fraction of inspired oxygen (FiO_2_), respiratory rate, systolic blood pressure, peripheral oxygen saturation, and Glasgow Coma Scale (GCS). Laboratory variables included haemoglobin and blood urea which are increasingly available in LMICs [20]. Treatment measures included use of mechanical ventilation, vasopressors, and antibiotics administered at the time of or immediately following admission. Diagnosis and co-morbidities were not included during model development, as internationally translatable (e.g. SNOMED, ICD-10) and reproducible diagnostic information is difficult to obtain outside research settings in LMICs.

Three versions of the model were proposed based on their applicability according to available resources: model 1 included clinical, laboratory, and treatment variables; model 2 included both clinical and laboratory variables; and model 3 included clinical parameters only. Treatment factors were only included in model 1 since these vary widely in relation to resource availability and local practices. Similarly, laboratory parameters were excluded from model 3 to evaluate a simpler and entirely clinical model which could be applicable to the most resource-limited settings, where seemingly simple blood tests such as haemoglobin and blood urea may be unavailable.

### Development of the predictive model

The distributions of the continuous variables were assessed, and skewed distributions were log normalized (e.g. blood urea) (Additional file 1: Figure S1). A univariate logistic regression model was fitted using the covariates listed above in order to assess their association with ICU mortality. The unadjusted estimates of the odds ratio for each variable was assessed and reported with *p* values and 95% confidence intervals (CIs). All variables independently associated with ICU mortality in the univariate model were then used to develop the predictive model. A stepwise backward-elimination procedure with probability of entry set at alpha = 0.05 and the probability of removal set at alpha = 0.055 was used to identify contributing variables. Collinearity was considered where thought plausible, e.g. between saturation and respiratory rate and between heart rate and respiratory rate. All analyses were performed using Stata software version 14.0 [21]. Statistical significance was set at 5% significance level (i.e. alpha = 0.05).

### Model validation

The three models thus obtained were validated in order to estimate their performance in similar populations. The potential for instability and reduced reliability of performance described as associated with splitting smaller medical datasets for model development and validation meant that bootstrapping was the favoured methodology for this study [22, 23, 24, 5]. A technique of bootstrapping (1000 samples) was utilized to enable a more precise representation of the population [5, 6]. The receiver operating characteristic (ROC) curve was obtained together with the C-Index (area under receiver operating characteristic curve (AUC)) with its 95% confidence interval. The sensitivity and specificity were calculated corresponding to the optimal cut-off probability of poor prognosis. Calibration (Hosmer Lemeshow C-Statistic) and accuracy of the model (Brier score) were also determined. Finally, the ability of each model to predict mortality in the most frequently occurring APACHE II diagnostic categories for the dataset was assessed.

### Comparison with existing prognostic models

The performance of the three models was then compared with APACHE II and SAPS II models [19]. APACHE II scores and probabilities were calculated for the datasets using the ICU admission parameters and the worst clinical and laboratory values from the first 24 h [19]. Discrimination (AUC), calibration (Hosmer-Lemeshow C-Statistic), and accuracy (Brier score) were calculated. The missing values were imputed as normal, according to common practice in HICs and as described in the original model publications [19, 25, 26].

### Nomogram development for the selected model

The model with the best overall performance and clinical applicability was translated into a nomogram using Stata statistical software [21]. The nomogram is intended as a graphical tool which can be readily used by clinicians, even in the absence of statistical software or online electronic calculators.

## Results

A total of 3855 patient records were available for analysis; 2283 patients were from India, 430 from Bangladesh, 325 Nepal, and 817 from 18 ICUs in Sri Lanka. Table 1 summarises the baseline characteristics of patients on admission to the ICU (Additional file 2: Table 1). Table 2 demonstrates that all the selected variables, except age, were significantly associated with ICU mortality in the univariate analysis. For age (Additional file 3: Figure S7) the difference in distribution between survivors (median 56, interquartile range (IQR) 37–68) and non-survivors (median 54, IQR 37–69) was not significantly different (*p* = 0.6276).

**Table 1:** Patient characteristics at the time of intensive care unit presentation described using APACHE II, SAPS II, and the new models

Variable	Nonsurvivors (*n* = 1031)	Survivors (*n* = 2590)	Availability *n* (%)
Body temperature (°C)^AII, SII^	37 (0.73)	37 (0.75)	3533 (91.6)
Heart rate^AII, SII^	109 (24)	100 (24)	3419 (88.7)
Age (years), median (range)^AII, SII^	54 (16–102)	56 (16–103)	3509 (91)
Respiratory rate^M1, M2, M3, AII^	24 (8)	23 (6)	2733 (70.9)
Systolic blood pressure (mmHg)^M1, M2, AII, SII^	132 (35)	139 (29)	2828 (73.4)
Diastolic blood pressure (mmHg)^AII, SII^	75 (19)	79 (16)	3527 (91.5)
Glasgow Coma Score, median (range)^M1, M2, M3, AII, SII^	9 (2–15)	15 (2–15)	3372 (87.5)
PaO_2_ (mmHg) median (range)^AII, SII^	150 (1.4–242)	150 (0.4–267)	423 (11)
pH^AII^	7.3 (0.15)	7.4 (0.13)	512 (13.3)
WBC^AII, SII^	21.3 (27.2)	18.6 (23.8)	2228 (57.8)
PCV %^AII^	34.7 (8.5)	35.8 (7)	463 (12)
Serum creatinine (mg/dL) median (range)^AII^	1.4 (0.3–11.3)	1.2 (0.4–18)	496 (12.9)
Potassium (mEq/L)^AII, SII^	4.2 (1.04)	4.1 (0.77)	2774 (72)
Sodium (mEq/L)^AII, SII^	137.7 (8.9)	137.1 (6.9)	2785 (72.2)
Blood urea nitrogen (mg/dL) median (range)^M1, M2, SII^	59 (0.6–411)	32 (0.9–672)	1936 (50.2)
Haemoglobin (g/dL)^M1, M2^	10.8 (2.8)	11.6 (2.4)	1869 (48.5)
Vasopressor use count (%)^M1^	572 (64.34)	1860 (91.31)	2951 (76.5)
Mechanical ventilation count (%)^M1, SII^	899 (87)	2054 (79)	2978 (77.3)
FiO_2_, median (range)^AII, SII^	35 (21–100)	21 (21–100)	2323 (60.3)
Urine output, median (range)^SII^	1150 (0–7400)	1300 (0–12000)	2469 (64)
Bicarbonate (mEq/L)^SII^	22.3(5.4)	22.5 (6.5)	361 (9.4)
Bilirubin (mg/dL) median (range)^SII^	1.1 (0.1–39)	0.7 (0.1–56)	1272 (33)

Values for nonsurvivors and survivors are given as mean (standard deviation) unless otherwise stated

Availability is described for the whole population

*AII* Acute Physiology and Chronic Health Evaluation (APACHE) II, *FiO*_*2*_ fraction of inspired oxygen, *M1* model 1, *M2* model 2, *M3* model 3, *PaO*_*2*_ partial pressure of oxygen, *PCV* packed cell volume, *SII* Simplified Acute Physiology Score (SAPS) II, *WBC* white blood cells

**Table 2:** Univariate analysis of associations between intensive care unit mortality and covariates

Covariate	Odds ratio	95% confidence interval	*p* value	*n*
Age	0.922	0.737–1.153	0.475	3621
Emergency surgery	1.933	1.559–2.397	0.000	3585
Temperature	1.435	1.298–1.586	0.000	3192
Systolic blood pressure	0.989	0.985–0.993	0.000	2828
Saturation	0.931	0.914–0.948	0.000	3621
Respiratory rate	1.024	1.011–1.038	0.000	2733
Heart rate	1.014	1.011–1.017	0.000	3419
Glasgow Coma Scale	0.844	0.829–0.858	0.000	3372
Fraction of inspired oxygen	3.212	1.759–5.863	0.000	3621
Haemoglobin	0.875	0.839–0.911	0.000	1869
Blood urea	1.007	1.005–1.008	0.000	1936
Vasoactive use	0.175	0.143–0.215	0.000	2955
Antibiotic use	0.292	0.226–0.377	0.000	2032
Mechanical ventilation	0.140	0.116–0.168	0.000	2948

Model 1 included the respiratory rate, systolic blood pressure, GCS, blood urea, haemoglobin, mechanical ventilation on ICU admission, and vasopressor use on ICU admission. Model 2, derived by excluding the resource-dependent treatment factors, included emergency surgery, respiratory rate, systolic blood pressure, GCS, blood urea, and haemoglobin. Model 3 was then derived by retaining only the clinical measures; respiratory rate, emergency surgery, and GCS were the parameters retained in the model after multivariable analysis. Systolic blood pressure was not significant in the model and remained not significant even when emergency surgery was excluded. The selected covariates for models 1, 2, and 3 and their coefficients are illustrated in Table 3.

**Table 3:** Multivariable logistic regression model of mortality on clinical and laboratory parameters

	Model 1 (*n* = 1125)		Model 2 (*n* = 1130)		Model 3 (*n* = 2069)	
Covariate	Beta-coefficient^#^	95% CI	Beta-coefficient^#^	95% CI	Beta-coefficient^#^	95% CI
Emergency surgery	0.437	–0.136, 1.010	0.547	0.025, 1.069*	0.730	0.439, 1.020*
Respiratory rate	0.060	0.018, 0.102*	0.005	0.002, 0.007*	0.064	0.041, 0.087*
Systolic blood pressure	–0.019	–0.031, –0.008*	0.002	0.001, 0.004*	0.003	–0.002, 0.008
Glasgow Coma Scale	–0.099	–0.139, –0.059*	–0.150	–0.185, –0.115*	–0.128	–0.151, –0.105*
Blood urea	0.006	0.003, 0.008*	0.006	0.004, 0.009*	NA	–
Haemoglobin	–0.093	–0.160, –0.027*	–0.098	–0.161, –0.034*	NA	–
Vasoactive use	1.057	0.5613, 1.5527*	NA	–	NA	–
Mechanical ventilation	1.429	0.9919, 1.8661*	NA	–	NA	–
Constant	6.164	4.374, 7.953	0.588	–0.358, 1.534	0.229	–0.061, 0.518

*The covariate was selected for the respective model

^#^The log of the odds ratio, i.e. the coefficient needed to calculate the model

*CI* confidence interval, *NA* not applicable

Only weak collinearity was shown between respiratory rate and saturation and between respiratory rate and heart rate (Additional file 4: Figure S2 and Additional file 5: Figure S3), which did not prompt modifications to the model.

Discrimination (AUC) for the three models were 0.818 (95% CI 0.800–0.835) for model 1, 0.767 (0.741–0.792) for model 2, and 0.725 (0.688–0.762) for model 3 (Table 4 and Additional file 6: Figure S4). The Hosmer Lemeshow C-Statistic had *p* values of less than 0.05 for models 1 and 3, but for model 2 (named the Tropical Intensive Care Score (TropICS)) it had a score of 11.3 and *p* value equal to 0.18, indicating statistically acceptable agreement between observed ICU mortality and ICU mortality. This would suggest that model 2 has good predictive ability [27]. The Brier score, denoting model accuracy in measuring prediction at an individual level, varied from 0.13 for model 1 to 0.18 for model 3, denoting acceptable performance for all three models [22]. The best sensitivity and specificity was achieved with model 1. In this model at the optimal cut-off probability for poor outcome (0.26), sensitivity was 71.9% and specificity 76.9%; these numbers were slightly lower for models 2 and 3 (Table 4).

**Table 4:** Performance of the three models and APACHE II and SAPS II

Performance	Model 1	Model 2	Model 3	APACHE II	SAPS II
Score, mean (SD)				17.35 (6.16)	
Probability, mean (SD)	0.20 (0.20)	0.20 (0.16)	0.28 (0.14)	0.26 (0.81)	0.15(0.19)
Optimal cut-off probability	0.18	0.18	0.26	0.2	0.13
Sensitivity (at optimum cut-off)	0.72	0.7	0.63	0.67	0.61
Specificity (at optimum cut-off)	0.77	0.69	0.67	0.71	0.72
AUC (95% CI)	0.812 (0.781–0.842)	0.767 (0.734–0.800)	0.689 (0.664–0.714)	0.707 (0.688–0.727)	0.714 (0.695– 0.732)
H/L C-statistic (*p*)	16.91 (*p* = 0.03)	11.31 (*p* = 0.19)	15.94 (*p* = 0.01)	124.84 (*p* < 0.01)	1692.14 (*p* = 0.000)
Brier score (95% CI)	0.13 (0.11–0.14)	0.14 (0.12–0.15)	0.18 (0.18–0.19)	0.18 (0.17–0.18)	0.20 (0.19– 0.21)

*APACHE* Acute Physiology and Chronic Health Evaluation, *AUC* area under the receiver operating characteristic curve, *CI* confidence interval, *H/L* Hosmer Lemeshow, *SAPS* Simplified Acute Physiology Score, *SD* standard deviation

**Fig. 1:**
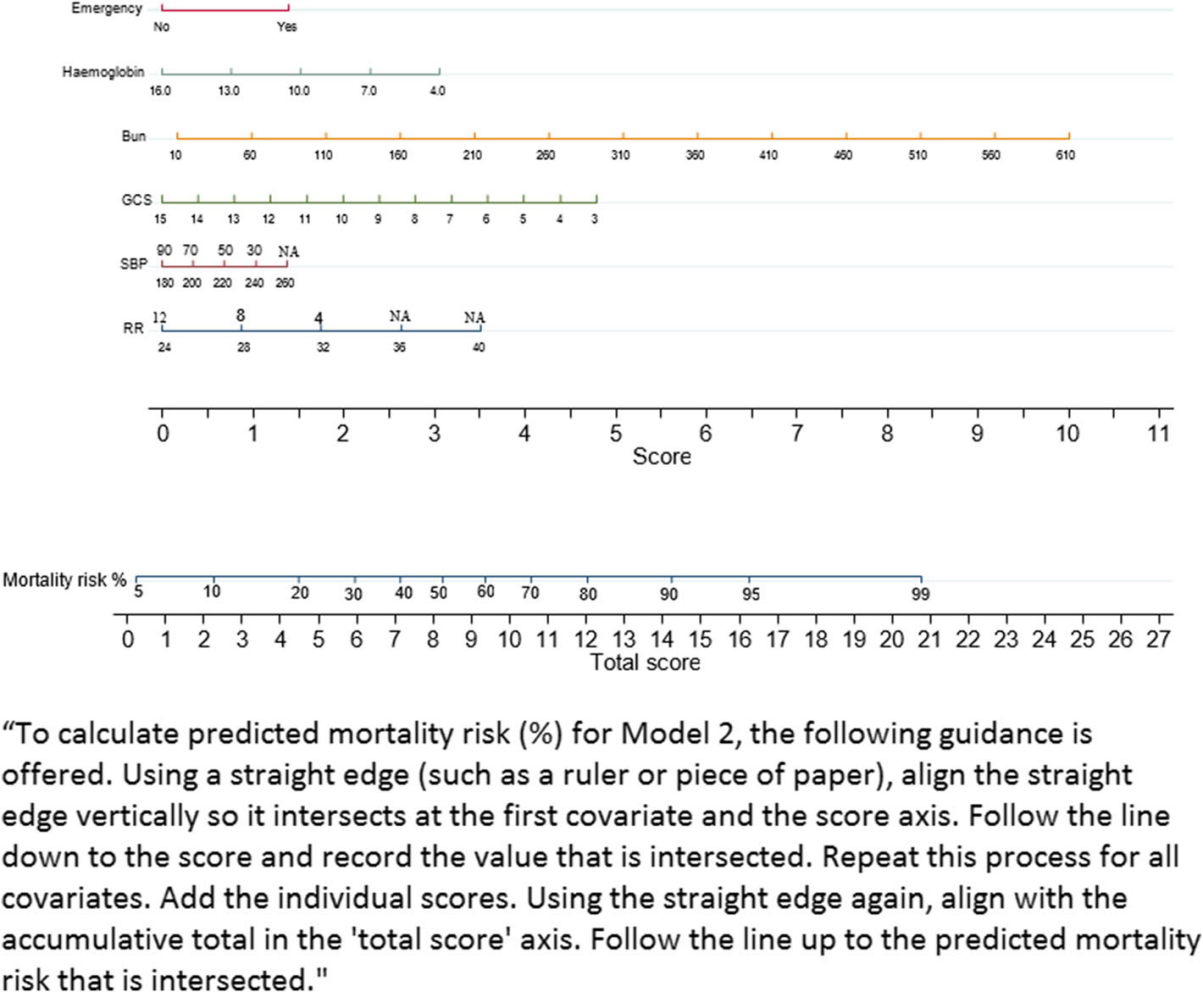
Nomogram for model 2. To calculate the predicted mortality risk (%) for model 2, the following guidance is offered. Using a straight edge (such as a ruler or a piece of paper) align the straight edge vertically so it intersects at the first covariate and the score axis. Follow the line down to the score and record the value that is intersected. Repeat this process for all covariates. Add the individual scores. Using the straight edge again, align with the accumulative total on the “total score” axis. Follow the line up to the predicted mortality risk that is intersected. *Bun* blood urea nitrogen, *GCS* Glasgow Coma Scale, *NA* not applicable, *RR* respiratory rate, *SBP* systolic blood pressure

In this dataset, the APACHE II model had an AUC of 0.707 (0.688–0.726) and SAPS II had an AUC of 0.714 (0.695–0.732). Calibration was very poor, with the Hosmer-Lemeshow C-Statistic being 124.84 (*p* < 0.01) and 1692.14 (*p* < 0.001) for APACHE II and SAPS II, respectively (Table 4). The ability of APACHE II, SAPS II, and models 1, 2 (TropICS), and 3 to predict outcomes in the commonest APACHE II categories in the dataset are shown in Additional file 7 (Figure S5). Additional file 8 (Figure S6) demonstrates the predicted versus actual mortality rates of the same models. The nomogram derived from model 2 for day-to-day clinical use is shown in Fig. 1.

## Discussion

This paper proposes a simplified, general critical care prognostic model developed for South Asia but with potential applications in LMICs worldwide. In this dataset, the model with clinical and laboratory parameters (model 2) and model 1, where the treatment characteristics were included, were both superior to APACHE II, SAPS II, and model 3, which was based on purely clinical variables. The authors propose the acronym TropICS (Tropical Intensive Care Score) for model 2 whose superior overall performance, simplicity, and objectivity, may enable prospective assessment in resource-limited settings in Asia, Africa, and South America to determine generalisability.

Critical care prognostic models are widely used in HICs for benchmarking, stratification of patients for research, and to assess quality improvement initiatives. Their applicability in LMICs is limited by the inclusion of relatively expensive laboratory parameters, diversity of case-mix, diversity of pathogenesis, the requirement for rigorous coding for diagnostic categorisation, and the difficulties in systematic data gathering in the absence of electronic records. The applicability and uptake of prognostics models in LMICs is poorly explored, in part due to limited availability of studies validating model performance. When such studies exist, the degree of missing information inhibits the generalisability of the evaluation [28, 29]. Existing models are infrequently used by clinicians, administrators, and decision makers in these settings, probably reflecting their perceived lack of relevance to the patient population and, in part, due to the lack of feasibility of data collection.

This relatively large and unique dataset from four South Asian countries enabled the development of a new prognostic model whilst attempting to address some of these described difficulties. This paper evaluates three levels of complexity during model development; model 1 uses clinical, laboratory, and treatment features, the second model uses only clinical and simple laboratory parameters (TropICS), whereas only clinical parameters are used in model 3.

Of the three proposed models, although models 1 and 2 had discrimination greater than 0.75, calibration was only adequate (*p* value = 0.19 > 0.05) for TropICS, suggesting the assignment of the correct probability at all levels of predicted risk [27]. In comparison with TropICS, both APACHE II and SAPS II performed less well with relatively poor discrimination (Table 4). Furthermore, both APACHE II and SAPS II had inferior accuracy and poor calibration in the same comparison. The APACHE II model performance is heavily affected by missing values and the techniques used for imputation [15]. In the current dataset sourced from diverse ICU settings in South Asia, the APACHE II complete case availability was only 15% (565 patients) with only approximately half the patients having haemoglobin and blood urea values. This illustrates that models developed for use in resource-limited settings must consider the availability and economic accessibility of obtainable variables. In addition, efforts should be encouraged to improve the measurement, accurate recording, and systematic data extraction, particularly in critical care settings. Improvement in data availability and recording can be expected from setting adapted medical registries (for example as recently implemented in Sri Lanka) and electronic patient information systems, especially if used in combination with simpler scoring systems and rapid clinician-led feedback mechanisms [17]. In addition, the importance of collecting data for such simple prognostic models should be emphasized by providing output that is relevant to clinicians, administrators, and patients. In addition, a setting-relevant model such as TropICS can be used for stratification of critically ill patients according to severity, which is a prerequisite for impact assessment of training and other quality improvement initiatives.

In the commonest APACHE II diagnostic categories, TropICS and model 1 performed best in predicting ICU mortality. The poor performance of prognostic scores developed in HIC when applied to diseases common in South Asia is a major shortcoming. The consistency of TropICS across the diverse diagnostic categories seen in the study settings is a positive finding for its possible generalisability. An important next step will be to assess the wider applicability of TropICS across further diagnostic categories.

Age, a covariate common in prognostic scoring models, was not retained in any of the proposed models and was not statistically significant between survivors and nonsurvivors (Additional file 1: Figure S7). Whilst widely accepted as a prognostic covariate, ICU prognostic validations conducted in both HICs and LMICs have reported limited predictive ability [30, 31]. Whilst an argument may have been made for its inclusion on purely clinical grounds, it was felt that further research is needed to understand the relationship with ICU mortality in non-HIC settings with younger patients being admitted to ICUs [32, 33, 34]. Similarly, no comorbidities were considered for model inclusion due to difficulties in ensuring uniform criteria for conditions such as chronic respiratory or renal disease in these settings. Additional parameters currently not included in the model warrant exploration; factors such as socioeconomic status, education levels, and access to healthcare may have greater impact on ICU outcomes in LMICs than in HICs.

TropICS can be used to predict ICU mortality, and the provided nomogram (Fig. 1) can be utilized for settings or circumstances where electronic devices may not be available; an online calculator will be hosted at www.nicst.com.

This study has several limitations. Despite using a minimal dataset, data collection was incomplete (Table 1), and the potential for bias cannot be excluded. In addition, external validation of the models in other LMICs is needed to assess generalisability and evaluate whether the differences in availability of data will affect model performance. ICU mortality was used as the endpoint in the development of the models, which may be influenced by nonclinical discharge decisions such as a shortage of ICU beds. Hospital or post-discharge mortality would be a more relevant outcome measure, and its unavailability is a major limitation.

## Conclusion

This paper proposes TropICS, the first international critical care prognostic model developed in non-HIC settings, for use in South Asian settings with potential for application in low- and middle-income countries worldwide. TropICS outperformed the APACHE II model in this South Asian dataset.

## Additional files

Additional file 1: Figure S1. Distribution of covariates of the TropICS model. (TIF 1564 kb)

Additional file 2: Table S1. Distribution of patient with chronic health conditions, as described for APACHE II model. (DOCX 13 kb)

Additional file 3: Figure S7. Distribution of age amongst survivors and nonsurvivors. (JPG 41 kb)

Additional file 4: Figure S2. Collinearity between heart rate and respiratory rate. (JPG 73 kb)

Additional file 5: Figure S3. Collinearity between saturation and respiratory rate. (JPG 61 kb)

Additional file 6: Figure S4. AUC for models 1, 2, and 3 and for APACHE II and SAPS II. (TIF 1564 kb)

Additional file 7: Figure S5. Model prediction—common APACHE II diagnoses. (TIF 89 kb)

Additional file 8: Figure S6. Predicted and actual mortality for each probability quartile for all models. (TIF 64 kb)

## Abbreviations

APACHE: Acute Physiology and Chronic Health Evaluation
AUC: Area under the receiver operating characteristic curve
CI: Confidence interval
GCS: Glasgow Coma Scale
HIC: High-income country
IQR: Interquartile range
LMIC: Lower- and middle-income country
SAPS: Simplified Acute Physiology Score
TropICS: Tropical Intensive Care Score
