# Supplementary figures and images for: Simplified prognostic model for critically ill patients in resource limited settings in South Asia
Source: Crit Care. 2017 Oct 17;21:250. doi: 10.1186/s13054-017-1843-6 (PMC5645891; doi:10.1186/s13054-017-1843-6)

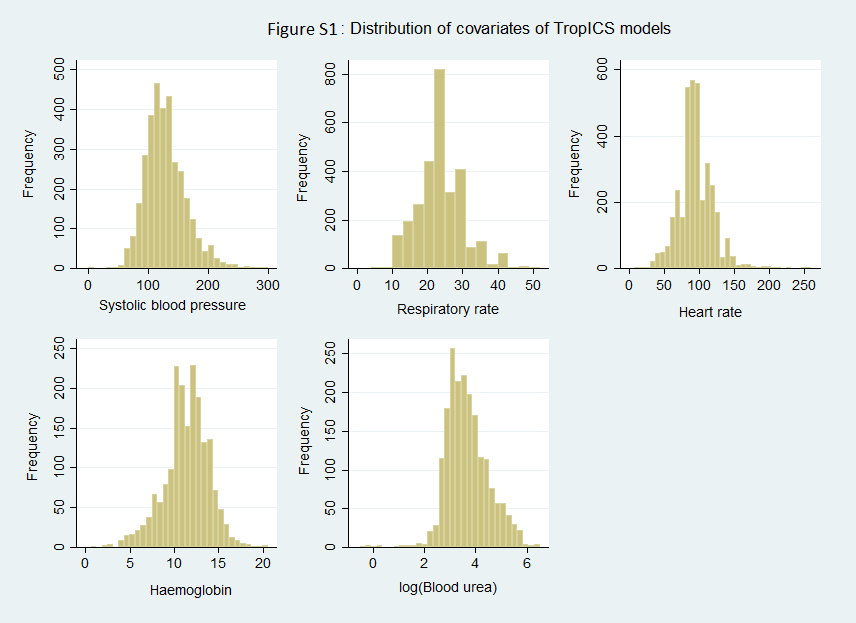

Supplement: Supplementary file 1 — Distribution of covariates of the TropICS model. (TIF 1564 kb) [file 13054_2017_1843_MOESM1_ESM.tif]

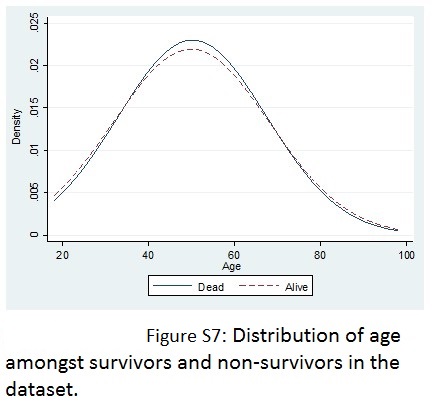

Supplement: Supplementary file 3 — Distribution of age amongst survivors and nonsurvivors. (JPG 41 kb) [file 13054_2017_1843_MOESM3_ESM.jpg]

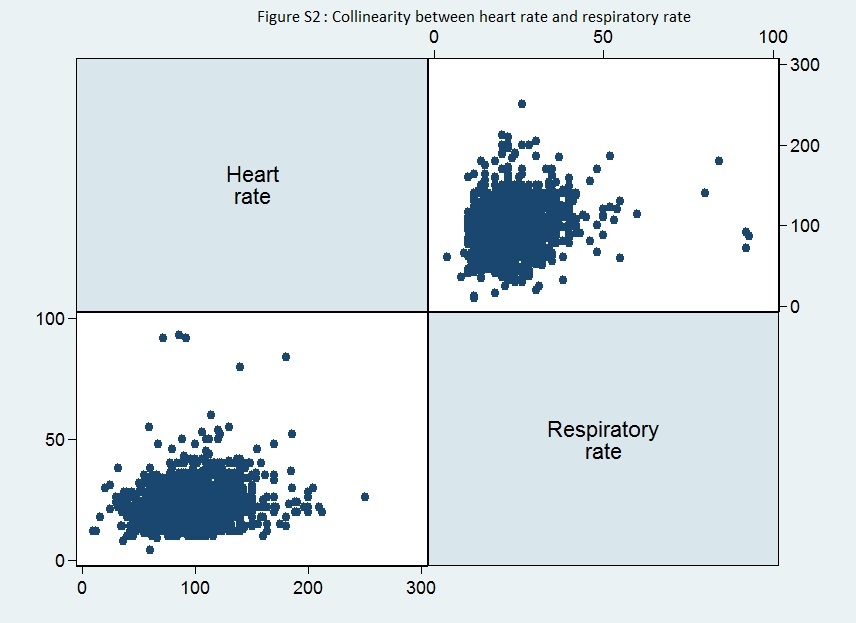

Supplement: Supplementary file 4 — Collinearity between heart rate and respiratory rate. (JPG 73 kb) [file 13054_2017_1843_MOESM4_ESM.jpg]

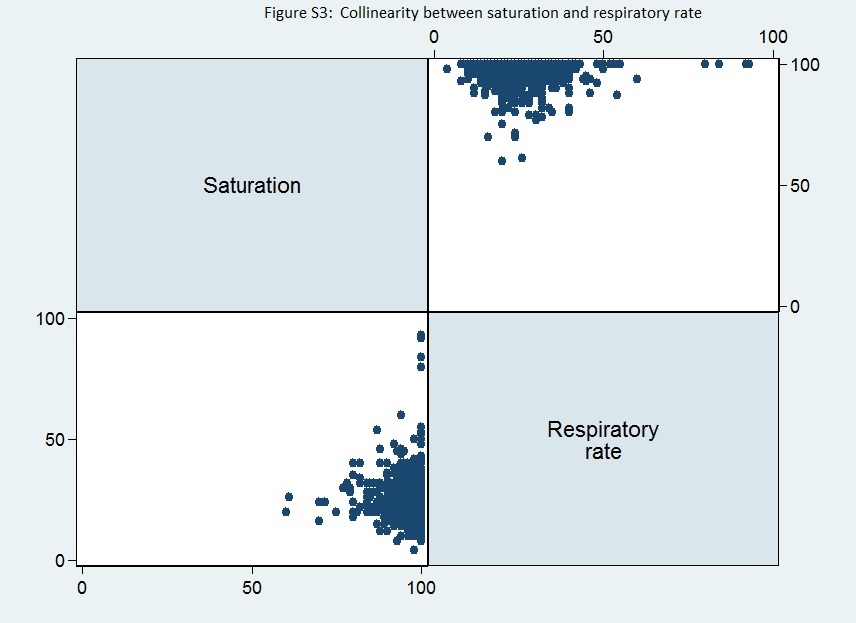

Supplement: Supplementary file 5 — Collinearity between saturation and respiratory rate. (JPG 61 kb) [file 13054_2017_1843_MOESM5_ESM.jpg]

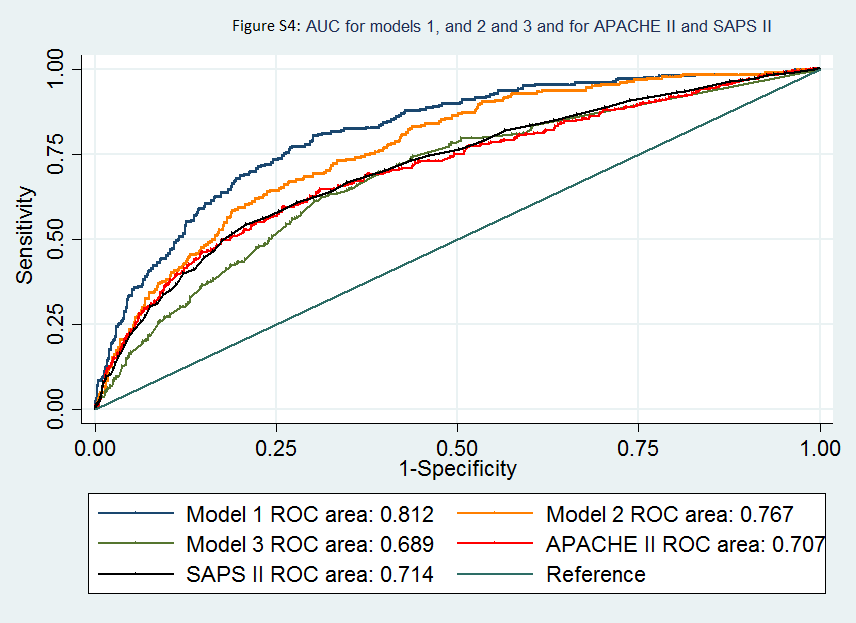

Supplement: Supplementary file 6 — AUC for models 1, 2, and 3 and for APACHE II and SAPS II. (TIF 1564 kb) [file 13054_2017_1843_MOESM6_ESM.tif]

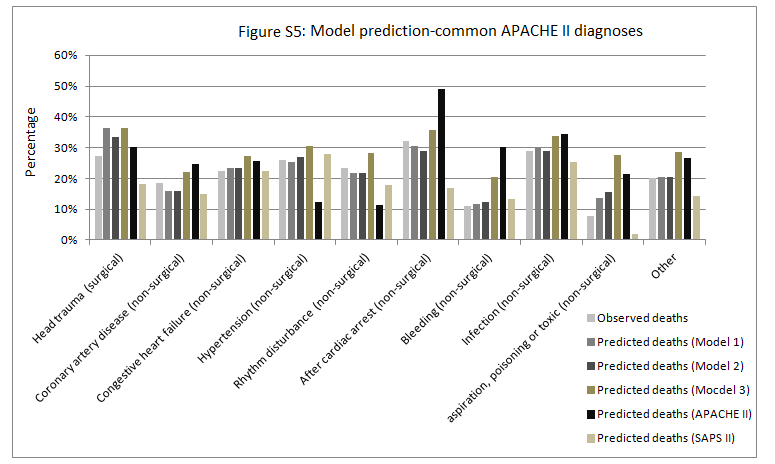

Supplement: Supplementary file 7 — Model prediction—common APACHE II diagnoses. (TIF 89 kb) [file 13054_2017_1843_MOESM7_ESM.tif]

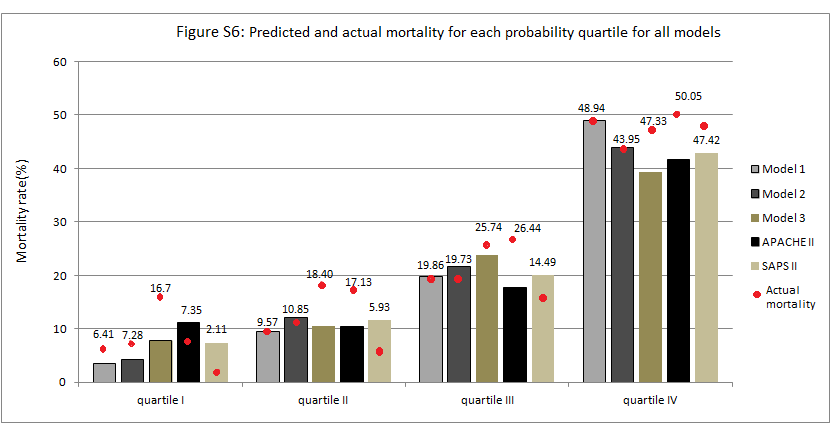

Supplement: Supplementary file 8 — Predicted and actual mortality for each probability quartile for all models. (TIF 64 kb) [file 13054_2017_1843_MOESM8_ESM.tif]
