# Supplementary material for: Simplified prognostic model for critically ill patients in resource limited settings in South Asia
Source: Crit Care. 2017 Oct 17;21:250. doi: 10.1186/s13054-017-1843-6 (PMC5645891; doi:10.1186/s13054-017-1843-6)
Supplement: Supplementary file 2 — Distribution of patient with chronic health conditions, as described for APACHE II model. (DOCX 13 kb) [file 13054_2017_1843_MOESM2_ESM.docx]

|  | **Non survivors (n=1031)** | **Survivors**  **(n=2590)** | **Availability**  **(%)** |
| --- | --- | --- | --- |
| **Gender** |  |  |  |
| Male | 657 (64.5) | 1653 (64.3) | 3786 (98.2) |
| **Admission type** |  |  |  |
| Medical | 804 (78.8) | 2163 (84.4) | 3775 (97.9) |
| Emergency surgery | 164 (16.1) | 231 (9) | 3775 (97.9) |
| Planned surgery | 52 (5.1) | 168 (6.6) | 3775 (97.9) |
| **Chronic health conditions** |  |  |  |
| Chronic renal insufficiency | 57 (5.7) | 79 (3.2) | 3646 (94.6) |
| Chronic respiratory insufficiency | 107 (10.6) | 151 (6) | 3658 (94.9) |
| Chronic dialysis | 15 (1.6) | 30 (1.3) | 3333 (86.5) |
| Chronic cardiovascular insufficiency | 40 (4.3) | 73 (3.2) | 3289 (85.3) |
| Diabetes | 200 (19.8) | 538 (21.4) | 3675 (95.3) |
| Cirrhosis | 12 (1.4) | 29 (1.4) | 2957 (76.7) |
| Immunological deficiency | 2 (0.2) | 3 (0.1) | 2953 (76.6) |

Table S1: Distribution of patient with chronic health conditions, as described for APACHE II model.
